# A neonate with spontaneous arterial limb ischemia and an aneurysm of the oval foramen: a case report

**DOI:** 10.1186/s13256-021-03078-9

**Published:** 2021-10-25

**Authors:** J. J. van Vonderen, J. M. H. Keus, J. van Schaik, F. J. Smiers, D. J. ten Harkel, E. Lopriore

**Affiliations:** 1 Division of Neonatology, Department of Pediatrics, Willem-Alexander Children’s Hospital, Leiden University Medical Center, Leiden, The Netherlands; 2 Department of Surgery, Leiden University Medical Center, Leiden, The Netherlands; 3 Division of Hematology and Stem Cell Transplantation Program, Department of Pediatrics, Willem-Alexander Children’s Hospital, Leiden University Medical Center, Leiden, The Netherlands; 4 Division of Cardiology, Department of Pediatrics, Willem-Alexander Children’s Hospital, Leiden University Medical Center, Leiden, The Netherlands

**Keywords:** Case report, Limb ischemia, Neonate, Aneurysm, Oval foramen

## Abstract

**Background:**

In this case report, we describe a very rare case of severe limb ischemia due to an arterial embolus caused by an aneurysm of the oval foramen in a term-born infant that occurred in the first few hours after birth.

**Case presentation:**

A newborn male Caucasian patient presented on the maternity ward with ischemia of the right foot. Ischemia was treated with nitroglycerin spray and low-molecular-weight heparin in therapeutic dosage. An aneurysm of the oval foramen was found during postnatal echocardiography screening. This was thought to be the source of an embolus causing limb ischemia. At birth and upon follow-up, no clotting disorders were found. A large part of the right forefoot was ischemic, leading to loss of digits 1, 2, and 3. No significant loss of function was found in the first year of life.

**Conclusion:**

Severe limb ischemia can be caused by an embolus arising from an aneurysm of the oval foramen and can be treated with heparin.

## Background

Spontaneous acute limb ischemia is a rare problem in neonates, occurring in 2.4 out of every 1000 patients admitted to the neonatal intensive care unit (NICU) [1]. It can have severe consequences and result in loss of limb. The vast majority of cases (88%) of acute limb injury in neonates are secondary to iatrogenic injury due to arterial cannulation and subsequent thrombosis [2–4]. Clotting disorders or vascular malformations can also be identified as causes, but there are only a few case reports of spontaneous ischemia without apparent cause [5]. We describe a case of an arterial embolus leading to ischemia of a limb that was most likely due to a thrombus originating from an aneurysm of the oval foramen.

## Case presentation

**Fig. 1:**
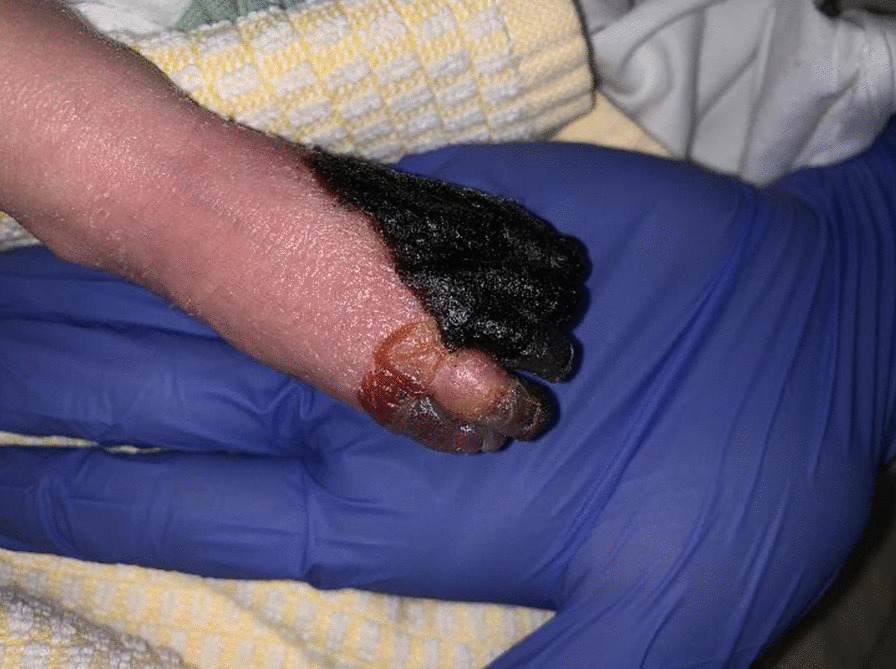
Right foot dorsal side after 2 days of life

**Fig. 2:**
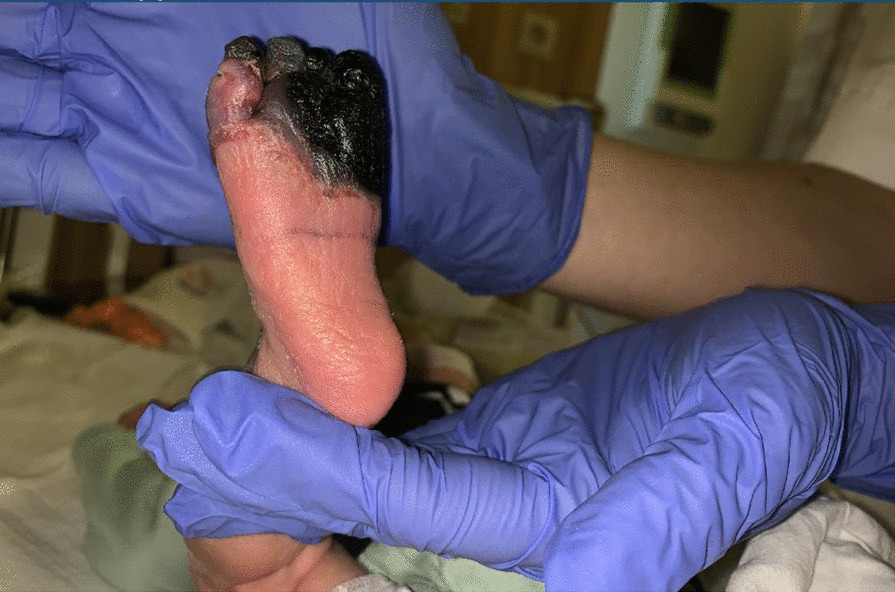
Right foot ventral side after 2 days life

During the first 4 hours after birth, a male neonate developed a sharply defined purple, painful, and swollen discoloration of the right foot. He was the second child of a dichorionic twin. The pregnancy had been uneventful, and the mother did not take any medication. Family history did not show any risk factors for clotting or bleeding disorders. At a gestational age of 37 weeks, delivery was induced because of intrauterine growth retardation of the second twin. This first child had an uneventful vaginal birth; however, due to weak contractions, the second child was born by cesarean section. There were no complications during his delivery (Apgar score 9/10/10), and no excessive force was used on his extremities during extraction. Physical examination at birth showed no abnormalities. Birth weight was 2980 g (p53). Approximately 3 hours after birth, the pediatrician was consulted because the right foot appeared to be less perfused, first showing sharply defined white discoloration and soon after turning purple, swollen, and painful. The neonate was immediately admitted to the NICU for monitoring. Repeated examination in the following hours showed purple discoloration on some distal phalanxes of the left hand. Nitroglycerine spray was applied to both extremities (Fig. 1). In the hours and days thereafter, the skin of the left forefoot became rapidly necrotic (Fig. 2).

## Investigations and differential diagnosis

Directly after birth, laboratory tests showed no coagulation disorders [activated partial thromboplastin time (APTT) 38.5 seconds, prothrombin time (PT) 14.6 seconds, fibrinogen 2.4 g/L], and there was no polycythemia (venous hematocrit 0.56 L/L). There were no laboratory signs of infections [C-reactive protein (CRP) 0.7 mg/L, normal full blood count, blood culture negative]. Furthermore, vital signs throughout clinical observation of the patient were unremarkable, and also blood gas was drawn upon admittance, which did not show any signs of a pulmonary cause of an embolism. Antibiotics (amoxicillin and gentamicin) were given for 48 hours, after which they were discontinued because no bacteria were cultured and infection values remained low, ruling out a septic thrombus.

Repeated Doppler ultrasound evaluation was done on day 1 and day 3, which showed patent femoral and crural arteries without a peripheral focus for an embolism.

**Fig. 3:**
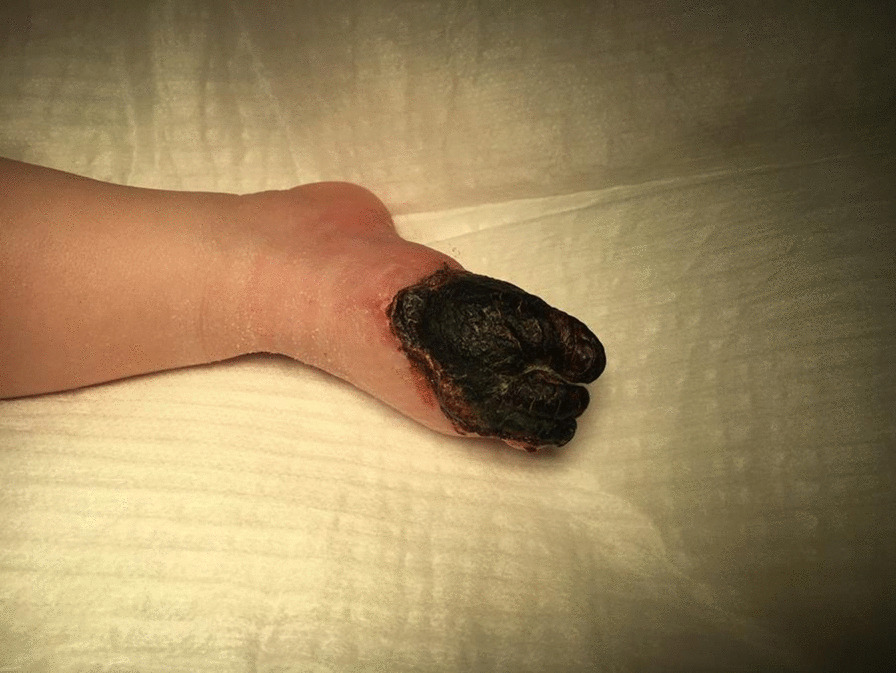
Right foot mummified after 1 week of life

The pediatric cardiologist was consulted to rule out cardiac thrombus. Repeated cardiac ultrasound revealed an aneurysm of the oval foramen without any vegetations or thrombus (Fig. 3). There was left-to-right shunt through the oval foramen. Cardiac ultrasound was otherwise normal.

**Fig. 4:**
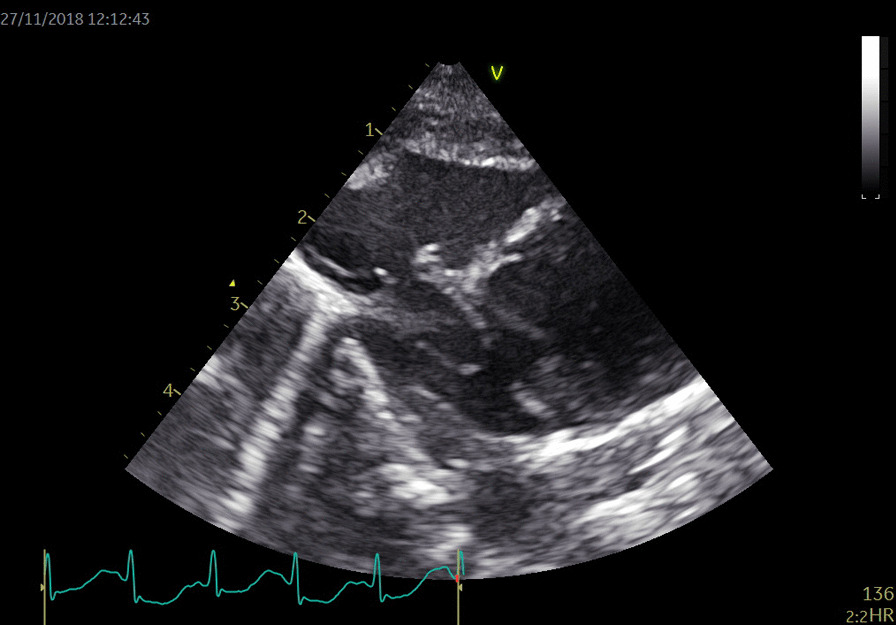
Ultrasound image of the septum with an aneurism of the oval foramen

In the weeks thereafter, digits 1, 2, and 3 and part of the proximal foot mummified (Fig. 4).

At 3 months of age, laboratory tests were repeated and remained negative for clotting disorders such as protein S and C deficiency.

The most probable cause of the limb ischemia was an arterial embolus originating from an aneurysm of the oval foramen as shown by cardiac ultrasound. This diagnosis is supported by the fact that the fingers of the left hand also showed signs of microemboli shortly after birth, suggesting a central source of embolization.

An iatrogenic cause was ruled out as no umbilical or arterial catheters were inserted. A septic embolus was unlikely since blood cultures were negative and infection parameters were low, 48 hours after which antibiotics were discontinued. Arterial vasoconstriction after birth was also unlikely since the birth was uneventful and there was no need for circulatory support. Polycythemia hyperviscosity syndrome was ruled out because of normal hematocrit.

## Treatments and outcome

The pediatric hematologist was consulted straight away. The hematologist had a preference for anti-thrombotic therapy with recombinant TPA (rTPA) together with low-molecular-weight heparin. Since there was only experience in the NICU with newborns treated with low-molecular-weight heparin, no rTPA was started, and heparin was chosen, which was administered in therapeutic dosage for 3 months. This was discontinued after repeated tests for clotting disorders appeared normal. After this time, treatment was continued with carbasalate calcium because the aneurysm of the oval foramen was still present.

Paracetamol and morphine were provided in the first 5 days after the thrombotic event had taken place as the leg was very painful at this time.

**Fig. 5:**
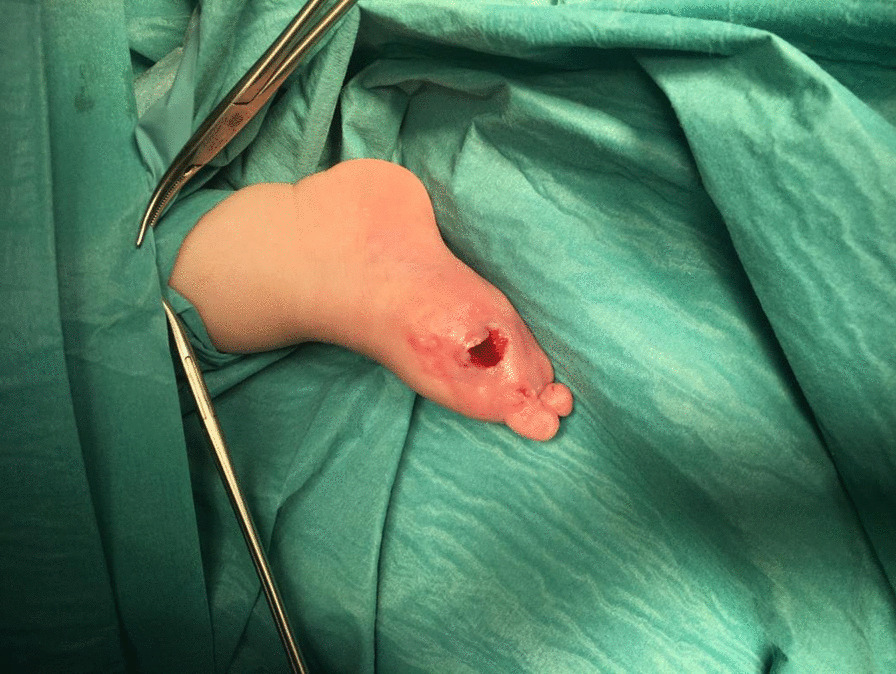
Right foot after operation at 3 months of age

The patient was followed up by the surgeon. After 3 months, digits 1, 2, and 3 of the right foot were completely mummified and were surgically removed without complications. There was no loss of function, and the patient has a similar development as his brother, reaching milestones adequately (Fig. 5). Cardiac follow-up was continued at 3-month intervals for a year, at which time the aneurysm disappeared.

## Discussion

Arterial limb occlusion is a rare event in otherwise healthy neonates without congenital abnormalities. Factors for spontaneous arterial limb occlusion include perinatal asphyxia, respiratory distress syndrome, maternal diabetes, neonatal sepsis, necrotizing enterocolitis, clotting disorders such as factor V Leiden, dehydration, congenital nephrotic syndrome, polycythemia, and twin–twin transfusion syndrome [6]. None of these risk factors was present in the case described. Since the investigations performed showed negative laboratory tests for clotting disorders and the only possible focus for an embolus was the aneurysm in the oval foramen, this was regarded as the most likely causal factor. This aneurysm may have caused multiple emboli leading to ischemia of the left hand and right foot. Cardiac aneurysm is a very rare finding in newborns and has been reported to lead to multiple emboli [7, 8]. Most of these aneurysms are treated conservatively with good outcome. Atrial septal aneurysms are localized saccular deformities of the interatrial septum generally at the level of the fossa ovalis, which bulges into the right or left atrium or both [9]. A literature search was performed, and to our knowledge, this was the first case in which a neonate suffered from an arterial embolus leading to limb ischemia likely arising from an aneurysm of the oval foramen.

Although the clotting system is overall balanced in term newborns, [10–12] it is known that the clotting system is very much activated during birth and in the days thereafter [13]. This prothrombotic status and the oval foramen aneurysm both probably contributed to the arterial embolus and ischemia.

There is no international consensus on the optimal management of occlusive events in neonates. Guidelines from the American College of Chest Physicians [14] recommend the use of either unfractionated heparin (UFH) combined with low-molecular-weight heparin (LMWH) or LMWH alone for 6-12 weeks after the incident. Thrombolysis with recombinant TPA (rTPA) is also suggested, but it has the potential to cause hemorrhage, which limits its use in neonates. In this case, we chose to use LMWH for 12 weeks as it is only administered once a day and is more commonly used in our institution. Whether the use of rTPA in this child would have prevented the loss of the three toes without the risk of bleeding remains unclear.

## Conclusion

In this case report, we describe a severe case of limb ischemia due to an arterial embolus in a term-born infant that occurred in the first few hours after birth. Ischemia probably originated from an embolus arising from an aneurysm of the oval foramen.

## Data Availability

The data that support the findings of this study are available on request from the corresponding author [JV]. The data are not publicly available due to them containing information that could compromise research participant privacy/consent.
